# A Comprehensive Study on the Hardening Features and Performance of Self-Compacting Concrete with High-Volume Fly Ash and Slag

**DOI:** 10.3390/ma14154286

**Published:** 2021-07-31

**Authors:** Zhenghong Yang, Sijia Liu, Long Yu, Linglin Xu

**Affiliations:** 1 Key Laboratory of Advanced Civil Engineering Materials, Tongji University, Ministry of Education, Shanghai 201804, China; yzh@tongji.edu.cn (Z.Y.); 1930662@tongji.edu.cn (S.L.); yulong@tongji.edu.cn (L.Y.); 2 School of Materials Science and Engineering, Tongji University, Shanghai 201804, China

**Keywords:** self-compacting concrete, fly ash, slag, hydration process, chloride permeability, pore structure

## Abstract

The main concern of this work is to evaluate the influences of supplementary cementitious materials (fly ash, slag) and a new type of polycarboxylate superplasticizer containing viscosity modifying agents (PCE-VMA) on the performance of self-compacting concrete (SCC). The workability, hydration process, mechanical property, chloride permeability, degree of hydration and pore structure of SCC were investigated. Results indicate that the addition of fly ash and slag slows down early hydration and decreases the hydration degree of SCC, and thus leads to a decline in compressive strengths, especially within the first 7 days. The addition of slag refines pore structure and contributes to lower porosity, and thus the chloride permeability of SCC is decreased during the late hydration stage. Additionally, a new factor of calculated water–binder ratio is put forward, which can directly reflect the free water content of concrete mixture after mixing, and guide the mix proportion design of SCC.

## 1. Introduction

Self-compacting concrete (SCC) has been widely applied to tackle the placement of congested reinforced concrete structures under difficult casting conditions [1]. High fluidity is the main characteristic of SCC, which can be casted without vibration, and segregation and bleeding during transportation or pouring could be significantly mitigated. Compared with traditional vibrated concrete, SCC could not only reduce the required time, labor and equipment on construction sites, but also improve the mobility of heavily congested structural elements. Hence, the noise and vibration-related injuries could be reduced effectively. Besides, SCC also produces high-quality-finished surfaces. However, to ensure the fluidity of SCC, more binders and chemical admixtures are necessary. Consequently, the cost of SCC is generally 20–50% higher than that of traditional vibrated concrete [2]. The high dosage of cement in SCC brings a high risk of rapid hydration heat releasing and considerable shrinkage, which threatens the volume stability and durability of concrete directly. In order to reduce this adverse effect, a high replacement level of supplementary cementitious materials (SCMs) has been confirmed as an effective solution.

The substitution of SCMs for cement has attracted much attention due to its positive effect on the overall performance of so-prepared concrete, and its sustainable development. High dosage of SCMs in SCC can reduce the cement consumption, hydration heat releasing and enhance the rheology of concrete mixture simultaneously. In particular, economical SCC has been successfully developed by incorporating high volumes of SCMs [2,3,4,5,6,7,8,9,10]. This trend has become more and more popular since SCC with a low mechanical grade such as C20 and C30 could easily satisfy the needs of normal constructions. The low grade SCC combines the advantages of both SCC and traditional ordinary concrete such as good stability, limited segregation and bleeding. However, SCMs would also induce decline of workability and durability of SCC. The addition of fly ash could broaden the size distribution of binders [11], thus ensuring greater cohesiveness [12]. The available studies [13,14,15,16,17,18] showed that the lower water requirement caused by the addition of fly ash could reduce the hydration heat releasing rate of cement and the chloride permeability, and promote later strength development. Moreover, the combination of slag and fly ash shows a synergic effect, especially for the compressive strength in the early stage [19]. This enables SCC to be applied to road concrete and marine concrete. In addition to SCMs, chemical admixture is another key component for SCC when a high deformation (low yield stress) and low bleeding and segregation (adequate viscosity) are required simultaneously [20]. Viscosity modifying agents (VMAs) offer a suitable solution for SCC with a high dosage of SCMs. VMAs are able to enhance the cohesion and stability of binders [21] through increasing water retention capacity and plastic viscosity at a given water–binder ratio [22]. On the other hand, VMAs are also effective in reducing bleeding due to the fact that the long-chain molecules of VMAs adhere to the periphery of water molecules. Furthermore, VMAs can absorb partially mixed water, which is able to increase the yield value and plastic viscosity of cementitious materials [23,24]. However, very limited information has been given about the influence of VMAs on the performance of SCC with high volumes of SCMs.

This paper presents a comprehensive study on the performance of SCC made with a high volume of slag and fly ash, and one type of polycarboxylate superplasticizer modified with VMAs. The hydration process was evaluated by calorimetry and non-evaporated water analysis. Physical properties such as workability, setting time, compressive strength and chloride permeability were determined in relation to the evolution of pore structure.

## 2. Experimental Procedure

### 2.1. Raw Materials

**Figure 1:**
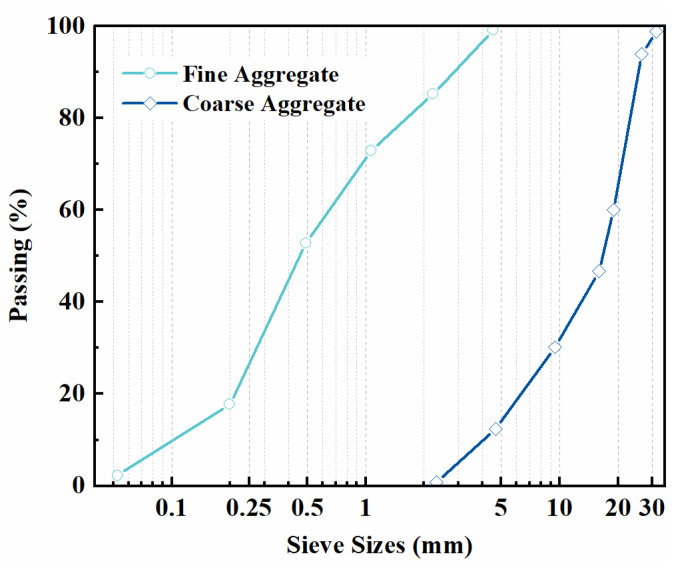
Particle size distribution of aggregates.

In this study, Portland cement (PII 42.5) and supplementary cementitious materials (SCMs), such as class F fly ash and grade S95 slag were used to prepare the binders. The specific gravity of cement, fly ash and slag are 3.2, 2.3 and 2.8, respectively, and the chemical composition are listed in Table 1. A new type of polycarboxylate superplasticizer (PCE, with solid content of 14.68%) modified with VMA (0.2 wt.% of PCE) was used in the mixtures. A naphthalene-based superplasticizer (NS, with solid content of 37.00%) was used as reference. Crushed basalt rock coarse aggregates with continuum grain sizes ranging from 5 mm to 31.5 mm were applied. River sand, which passed into a 4.75 mm sieve, was used as fine aggregate. The grading curves of aggregates are shown in Figure 1.

### 2.2. Mix Proportions and Preparation of Specimens

Mix proportions used in this study are summarized in Table 2. The mix proportion was selected to achieve a target compressive strength of 20 MPa at 28 days. C1, C2, C3, C4 and C5 represent the concrete specimens, and the corresponding binder paste specimens without aggregates were referred as P1, P2, P3, P4 and P5.

In this work, the total dosage of binders was kept at a constant of 380 kg/m^3^ for all mixtures and a water–binder ratio of 0.45 was used. Two kinds of SCMs, including fly ash and slag, were considered as substitutes for cement, and the replacement level of SCMs was kept equal to 47% in all mixtures by weight. The pure Portland cement without fly ash and slag was prepared as control (C5). The utilization percentage of superplasticizer represents the weight percentage of the superplasticizer in the binder.

Concrete mixture was cast in molds in one layer after mixing without any compaction. All the specimens were sealed with nylon sheets and stored under ambient room conditions for 24 h. The specimens were demolded after 24 h and cured under ambient conditions at 20 °C and 60 ± 5% relative humidity until the designed testing age.

### 2.3. Methods

The hardened binder paste was crushed and soaked in absolute ethanol to stop hydration. Then it was dried, ground and sieved through a 0.075 mm sieve to test the specific gravity. The workability of the fresh SCC mixtures was evaluated via the slump and slump flow tests according to ASTM C143.

In order to investigate the early hydration process of paste mixed with various SCMs, the heat releasing rate of mixed paste within the first 72 h at 20 ± 0.1 °C was monitored using calorimetry analysis (TAM air C80, Thermometric, Örebro, Sweden). Binders and water were stored at 20 ± 0.1 °C for several hours before mixing, and then the water was injected into the reaction vessel. The samples were stirred rapidly outside the calorimeter for 30 s.

A total of 15 cubic specimens with a size of 100 mm × 100 mm × 100 mm for each mix proportion were manufactured for compressive strength measurement. A universal testing machine with a loading rate of 0.5 MPa/s was used to determine the compressive strength according to GB/T 50081-2019. After being cured for 1, 3, 7, 28 and 56 days, three specimens were randomly selected for testing, and the final results are given from the average values and error bars.

The chloride migration tests were carried out on 3 cylinder specimens with a size of Ø100 × 50 mm at the age of 28 and 56 days. The test was performed according to ASTM C1202, in which the cylinders were firstly vacuum saturated with water for 24 h. Then, a 60 V DC external potential was applied on the specimens for 6 h, forcing the chloride ions to migrate from 3.0 wt.% NaCl solution into specimens. The total charge passed during 6 h was then determined in coulombs, which was related to the ability of concrete to resist chloride ion penetration.

The non-evaporable water content (*W_n_*) was calculated by the mass change of binder paste, as shown in Equation (1). The paste powder sample was dried at 60 °C until it reached a certain weight and then heated in an oven at 950 °C.

(1) $$W_{n}=\frac{W_{1}-W_{2}}{W_{2}}$$

where *W_1_* and *W_2_* denote the weight of binder paste before and after ignition, respectively.

Automatic mercury porosimeter (Thermal Scientific PASCAL 140/440 series) was applied to determine the pore structure of hardened paste. The mercury intrusion and extrusion were performed with an applied pressure initiated from 1.4 kPa to 240 MPa. The measurable pore size was in the range from 3.0 nm to 800.0 μm according to the Washburn equation, as shown in Equation (2). By continuously changing the applied pressure, the amount of mercury entering the pores can be measured and the pore size distribution can be obtained.

(2) Pr=−2γcosθ

where *Pr* is the applied pressure, *θ* is the contact angle between mercury and the solid phase and *γ* is surface tension. *θ* is 0.480 N/m and the *γ* of 140° is used.

## 3. Results and Discussion

### 3.1. Workability

**Figure 2:**
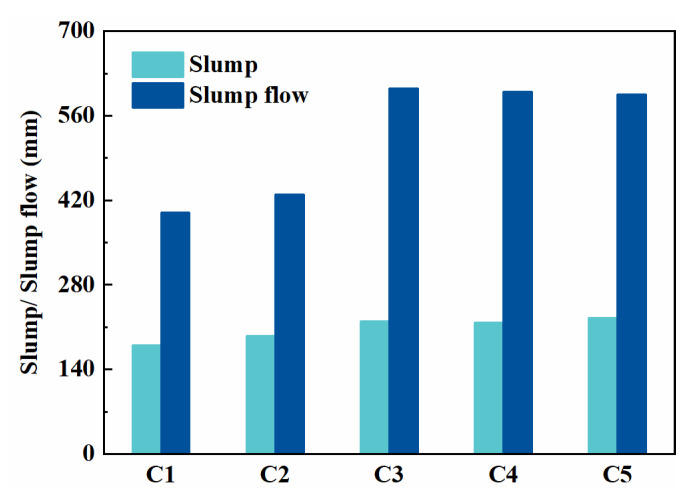
Slump/slump flow of SCC mixtures.

The workability of fresh concrete mixtures is one of the key technique items for SCC. As presented in Figure 2, the slump had no obvious relationship with the mix proportion of concrete. The slump and slump flow of the mixtures mixed with PCE-VMA were slightly greater than that with NS, which means that the PCE-VMA could achieve higher water reduction and better flow capability.

It can be found that the replacement of cement by fly ash or slag alone exerts no obvious effect on the slump and slump flow of SCC mixtures. But the combination of slag and fly ash resulted in a decreased slump flow of SCC. Spherical shaped particles of fly ash acted as micro beads to promote the flowability of the mixtures [25,26,27,28]. However, fly ash and slag showed different particle shapes and sizes, the sliding friction within the binder paste increased, which led to the decrease of slump flow.

The initial and final setting time of SCC mixtures are presented in Table 3. The final setting time of specimen C1 was 620 min, which was 35 min longer than that of C2. The initial setting time also showed a similar trend. The composition of cementitious material has more obvious effects on setting time than the type of superplasticizer. The addition of fly ash or slag alone prolonged the setting time, especially for those specimens with slag. It was inferred that part of the slag powder was absorbed and wrapped around the surface of cement particles, which delayed the hydration rate of cement particles [29].

### 3.2. Hydration Heat

**Figure 3:**
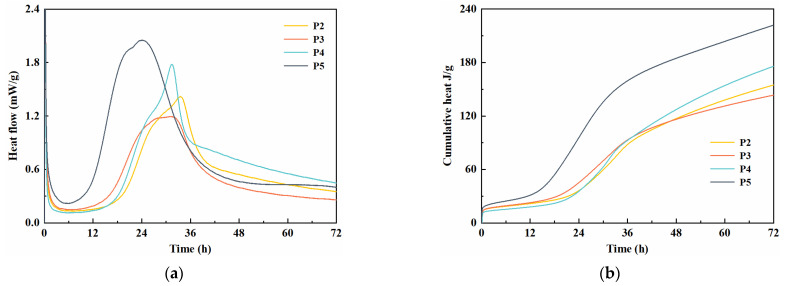
Heat release of binder pastes within 72 h. (**a**) rate of heat evolution; (**b**) cumulative hydration heat.

Figure 3 shows the effect of fly ash and slag on the heat flow rate and cumulative heat releasing rate of cement hydration during the first 72 h. As seen in Figure 3, it was apparent that specimen P5, which was prepared without SCMs, reached its main hydration peak at 24 h. The hydration-entered dormant period after the first exothermic peak, resulting from the concentration of Ca^2+^, required a certain time to reach saturation to boost further hydration [30,31].

It is found that specimen P5 showed the highest hydration exothermic reaction intensity and cumulative heat releasing rate, followed by specimens P4, P2 and P3. The replacement of cement by SCMs led to a significant reduction of release peak value and an extension time to reach the main peak, which indicated that SCMs could delay the early hydration of cement. This is due to the great reduction in the proportion of cement clinker in the binder with a large volume of SCMs. In addition, massive slag absorbed Ca^2+^ generated by cement hydration and generated CSH gel by pozzolanic reaction, leading to a low concentration of Ca^2+^, and thus delaying the nucleation and growth of hydration products [32,33,34].

As observed from Figure 3a, specimen P4 exhibited a higher exothermic reaction intensity than that of P2 and P3 around 32 h and a higher cumulative heat releasing rate after 36 h, confirming the latent hydration capacity of slag. The cumulative heat releasing rate of specimen P4 within the first 72 h was 175 J/g, which was 13.6% and 22.4% higher than those of specimens P2 and P3, respectively. This finding indicates that fly ash has a smaller specific surface area than slag, contains more SiO_2_ and Al_2_O_3_ networks, and the structure of its glass network is more stable. The hydration activity of fly ash is relatively low. Hence, the hydration heat of paste was greatly reduced by the addition of fly ash.

### 3.3. Compressive Strength

**Figure 4:**
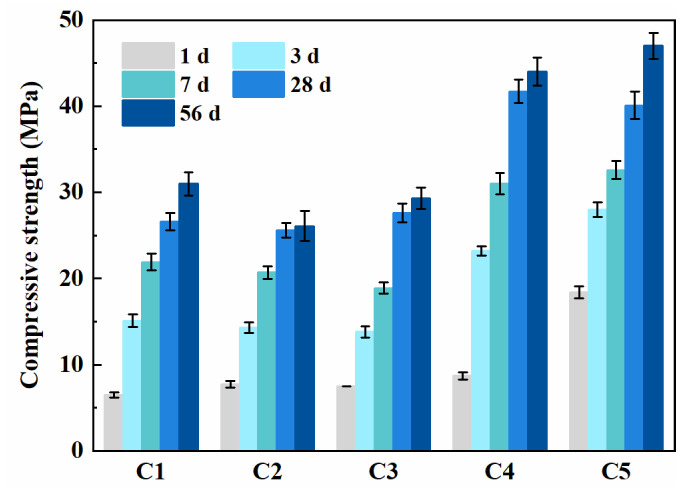
The compressive strength of SCC.

The compressive strength of SCC at the ages of 1, 3, 7, 28 and 56 days is presented in Figure 4. The compressive strength of all SCC reached the specified design of 20 MPa at 28 days. Compared with specimen C1, C2 only showed a slight improvement in 1-day strength and a comparable strength until 28 days, but a slight reduction at 56 days. This may lie in that PCE-VMA contributes to initial strength to some extent but is not conducive to the development of later strength.

Figure 4 shows that fly ash and slag reduced the compressive strength of SCC at all curing ages. The compressive strength of the specimens C2, C3 and C4 cured for 56 days only accounts for 55.5%, 66.6% and 93.6% of C5, respectively. The strength development of SCC with SCMs is slower than the control. In the case of specimen C4, the compressive strength increased more significantly than that of C3 after 28 days of curing. This is consistent with the results given by Kuder et al. [35].

Due to the different specific gravity of cement, fly ash and slag, the demand for water is different. Therefore, the actual free water content of the paste containing different kinds of SCMs is different. The calculated water–binder ratio (referred as *w*/b*) which reflects the free water content of the mixture after mixing can be put forward firstly in this present paper and calculated using the following equation:

(3) $$SG_{paste}=\frac{m_{binder}+m_{binder}\times \left(\frac{w}{b^{*}}\right)}{\frac{m_{cement}}{SG_{cement}}+\frac{m_{slag}}{SG_{slag}}+\frac{m_{fly\;ash}}{SG_{fly\;ash}}+m_{binder}\times \left(\frac{w}{b^{*}}\right)}$$

(4) $$\frac{w}{b*}=\frac{\left(\frac{m_{cement}}{SG_{cement}}+\frac{m_{slag}}{SG_{slag}}+\frac{m_{fly\;ash}}{SG_{fly\;ash}}\right)\times SG_{paste}-m_{binder}}{m_{binder}-m_{binder}\times SG_{paste}}$$

where *m_i_* means the mass of *i*, and $m_{binder}=m_{cement}+m_{slag}+m_{fly\;ash}$, and *SG_i_* stands for the specific gravity of *i* component. In this way, the calculated w/b ratio of C1, C2, C3, C4 and C5 specimens were 0.29, 0.25, 0.39, 0.31 and 0.39, respectively. Although specimens C3 and C4 have the same designed w/b as shown in Table 2, a different calculation of w/b reflects the free water content for each mixture after mixing. Specimen C3, with fly ash, has a calculated w/b of 0.39, much higher than that of C4 at 0.31. This is consistent with the fact that the compressive strength of specimen C3 is lower than that of C4. For SCC with different SCMs, the calculated w/b can be calculated according to the specific gravity of cement and SCMs, so as to adjust the actual water consumption. Additionally, the development of early strength is congruent with hydration heat. Higher hydration rate leads to a faster compressive strength development.

### 3.4. Chloride Permeability

**Figure 5:**
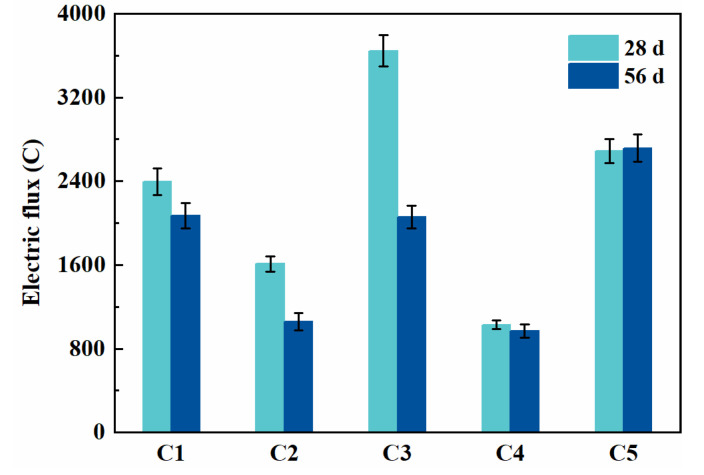
Electric flux of SCC at 28 and 56 days.

The electric flux measured here characterizes the chloride permeability of SCC, as shown in Figure 5. It can be found that chloride permeability was highly dependent on the mix proportion. The electric flux of C1, C2, C3, C4 specimens at 56 days were 2071 C, 1060 C, 2058 C, 969 C, which were 23.7%, 61.0%, 24.2% and 64.3% lower than that of C5, respectively. Among these five groups, specimen C4 exhibited the lowest electric flux after 28 and 56 days. The electric flux of specimen C3 was the highest at 28 days, and decreased prominently after 56 days. In general, fly ash had a positive effect on the chloride resistance of SCC later, while slag contributed throughout the whole testing period. This is different from traditional vibrated concrete. Meanwhile, it is known that fly ash and slag can both decrease chloride diffusivity due to their ability to refine the pore size distribution via pozzolanic reaction or latent hydration [36]. In addition, the electric flux of specimen C2 is 32.8% and 48.8% lower than that of C1 at 28 and 56 days, indicating PCE-VMA was beneficial to improving the chloride resistance of SCC in the long term.

### 3.5. Degree of Hydration

**Figure 6:**
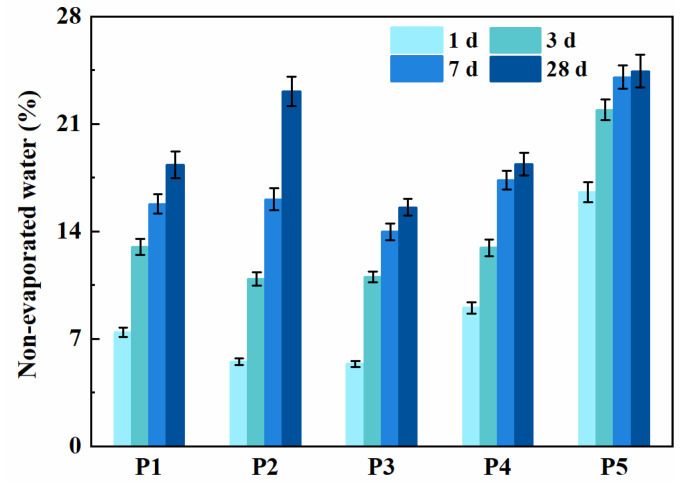
Non-evaporated water of binder pastes at 1, 3, 7 and 28 days.

The results of the non-evaporated water content (*W_n_*) of all binder pastes are presented in Figure 6. The non-evaporated water content depends on the type of hydration products employed. The degree of hydration reaction of binder paste is determined by the amount of hydration products used. Therefore, the non-evaporable water content can be used to infer the degree of hydration reaction. For specimen P5, the non-evaporated water content at 1, 3, 7 and 28 days were 16.6%, 21.9%, 24.0% and 24.4%. It can be seen that the hydration degree first significantly increased from 1 to 3 days, and then its increasing rate gradually decreased from 3 to 28 days. However, the non-evaporated water content of specimen P2 at 1, 3, 7 and 28 days were 33.3%, 49.7%, 66.9%, and 94.6% of P5, respectively. The increase rate of non-evaporated water content after 7 days seems to be higher than 1–3 days due to the pozzolanic effect of fly ash and slag.

For P2, P3 and P4 specimens, the non-evaporated water contents at 28 days were 23.1%, 15.6% and 18.4%, which were 94.6%, 63.9% and 75.4% of P5. It indicates that paste mixed with slag has a higher non-evaporated water content and higher degree of hydration than that with fly ash. Additionally, the non-evaporated water content of specimen P1 was slightly higher than that of specimen P2 after 3 days. It can be concluded that the varieties of superplasticizer play no prominent chemical contribution to early hydration. However, specimen P2 showed a higher hydration degree at a later stage.

### 3.6. Pore-Structure

**Figure 7:**
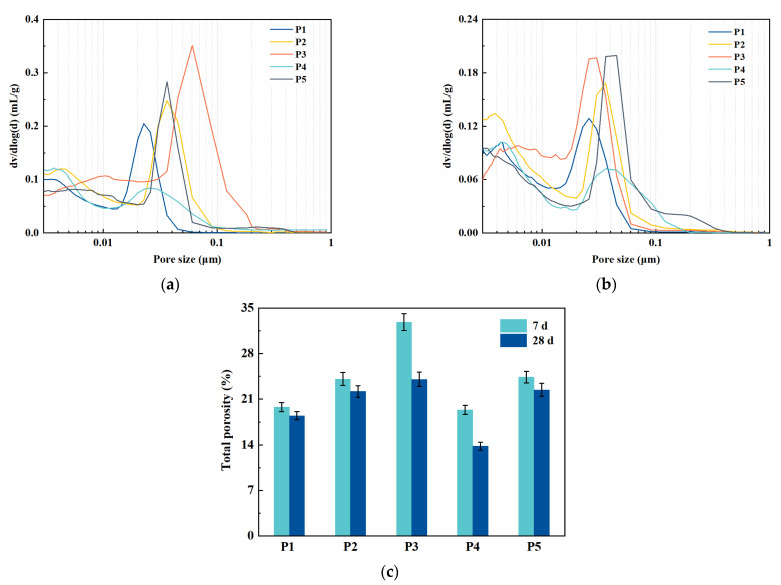
Pore structure of binder pastes: (**a**) pore size distribution, 7 days (**b**) pore size distribution, 28 days (**c**) total porosity.

Figure 7 presents the pore structures of binder paste specimens at the age of 7 and 28 days. Compared with specimen P5, the addition of fly ash increases the volume of pores with diameters ranging from 0.03 to 0.1 μm and the total porosity at both 7 and 28 days. However, the addition of slag presents an opposite influence on the pore structure. The refinement of pores in specimen P4 can be mainly attributed to the latent hydration of slag and the filling of the internal pores. The corresponding paste mixed with PCE-VMA shows more harmful pores and a higher porosity in comparison to specimen P1 and P2, which was consistent with the compressive strength measured (as can be seen in Figure 4).

In all binder pastes, increasing age from 7 to 28 days caused a great reduction in the total porosity. For instance, the total porosity of P3 was 24.1% at 28 days, lower than that at 7 days (32.9%) (Figure 7c). This illustrates the higher hydration degree of binders, especially for paste with SCMs.

## 4. Conclusions

The influence of fly ash, slag and PCE-VMA on the hydration process and physical properties of SCC were investigated. Some main conclusions can be drawn as follows:

(1) Slag showed an obvious accelerating impact on the early hydration and the degree of hydration of SCC than that of fly ash. From the perspective of pore structure development, the addition of fly ash resulted in more harmful pores within 7 days, while slag refined pore structure and contributed to lower porosity. SCC mixed with slag showed higher compressive strength and faster strength development at different ages than with fly ash.
(2) SCC mixed with PCE-VMA showed a lower compressive strength and higher porosity than with NS at 7 days and 28 days. However, the electrical flux of SCC mixed with PCE-VMA was 32.8% and 48.8% lower than that with NS at the age of 28 days and 56 days. Although PCE-VMA was not conducive to strength, it is beneficial to improve the later hydration degree and the chloride permeability.
(3) Additionally, instead of the designed water–binder ratio, the calculated water–binder ratio of sieved paste which could reflect the free water content of SCC mixture was more relevant. Consequently, the calculated water–binder ratio can also be taken into consideration as an important evaluation factor for SCC.

## Figures and Tables

**Table 1:** Chemical composition of raw materials (wt./%).

Raw Materials	Al_2_O_3_	SiO_2_	CaO	SO_3_	Na_2_O	K_2_O	MgO	P_2_O_5_	TiO_2_	Fe_2_O_3_
Cement	7.01	24.80	57.1	2.43	-	2.11	0.96	0.04	0.29	3.14
Fly ash	22.80	46.40	5.76	0.40	0.68	3.01	1.12	0.25	0.85	6.15
Slag	13.00	28.30	40.3	2.67	0.28	0.27	7.74	-	0.79	0.78

**Table 2:** Concrete mix proportions (kg/m^3^).

No.	Cement	Slag	Fly Ash	Fine Aggregate	Coarse Aggregate	Water	Superplasticizer	
							Type	Percentage (%)
C1	200	80	100	812	1033	171	NS	1.00
C2	200	80	100	812	1033	171	PCE	0.8
C3	200	-	180	812	1033	171	PCE	1.30
C4	200	180	-	812	1033	171	PCE	1.40
C5	380	-	-	812	1033	171	PCE	1.60

**Table 3:** The setting times of SCC mixtures.

No.	Initial Setting (min)	Final Setting (min)
C1	490	620
C2	435	585
C3	630	760
C4	750	870
C5	450	540

## Data Availability

The data presented in this study are available from the corresponding author upon reasonable request.
